# Contrastive learning-enhanced personalized interaction dual tower network for recommendation

**DOI:** 10.1371/journal.pone.0332894

**Published:** 2025-10-23

**Authors:** Fang Yang, Binghui Wang, Pengliang Li

**Affiliations:** 1 Malaysia SEGi University, Kuala Lumpur, Malaysia; 2 Handan Congtai Wine Company Limited, Handan, China; King Fahd University of Petroleum & Minerals, SAUDI ARABIA

## Abstract

Dual-tower retrieval models have become a prevalent solution in large-scale recommendation systems due to their scalability and deployment efficiency. However, they face critical limitations including insufficient modeling of user behavior sequences, lack of personalized inter-tower interactions, and poor representation learning for long-tail content. To address these issues, we propose a novel framework called Contrastive Learning-Enhanced Personalized Interaction Dual Tower Network (CL-EPIDTN). This model integrates a multi-layer Transformer to capture dynamic user preference shifts, and introduces a dual-path personalized enhancement mechanism to strengthen user–item feature dependencies. Additionally, a contrastive learning strategy is employed to enhance the representation learning of long-tail items and low-activity users under sparse data conditions. Extensive experiments on two public datasets (Amazon Books and TmallData) demonstrate the effectiveness of our method. CL-EPIDTN achieves the best performance across multiple metrics, with Hit Rate@10 of 0.0351 and Recall@50 of 0.1123 on Amazon Books, and Hit Rate@10 of 0.0901 and Recall@50 of 0.1599 on TmallData, outperforming six state-of-the-art baselines. These results highlight the potential of CL-EPIDTN for both academic research and practical deployment in real-world recommender systems, particularly in handling personalization and data sparsity challenges.

## 1. Introduction

With the explosive growth of online content and user engagement, large-scale recommendation systems have become a vital tool in helping users efficiently discover relevant information [1]. In such systems, the recall stage plays a critical role by narrowing down the vast item pool into a manageable candidate set for subsequent ranking. To meet the demands of low latency and scalability at this stage, dual-tower retrieval models have emerged as the mainstream architecture [2]. These models project users and items into a shared embedding space via two separate neural networks (towers), and use vector similarity to estimate user–item relevance. Due to their efficiency and compatibility with real-time inference, dual-tower architectures are widely adopted in industrial recommendation platforms [3].

Despite these advantages, conventional dual-tower models exhibit critical limitations when applied in sparse-data and personalized recommendation scenarios [4]. First, they typically rely on static user representations derived from historical features, overlooking the sequential dynamics of user behaviors and thereby failing to capture evolving preferences. Second, the two towers usually operate independently, with insufficient personalized interaction mechanisms to align user and item representations. Third, real-world systems often involve a large proportion of long-tail items and low-activity users, for which the models struggle to learn robust embeddings due to limited interaction signals [5,6]. For example, if a user recently develops an interest in a niche domain such as historical documentaries after primarily consuming mainstream action content, a conventional dual-tower model is likely to persist in recommending action-oriented items. Moreover, long-tail items within the newly emerging interest domain may be neglected entirely due to data sparsity [7,8]. To overcome these challenges, recent research highlights the potential of contrastive learning for recommendation. From a theoretical perspective, contrastive learning constructs informative training signals by contrasting positive and negative pairs, even when explicit interactions are sparse [9]. This approach not only enhances the clustering of semantically similar long-tail items in the embedding space but also provides auxiliary objectives that regularize training and mitigate overfitting to popular head classes [10,11]. Nevertheless, integrating contrastive learning into dual-tower frameworks remains underexplored, particularly in balancing efficiency with fine-grained personalization.

This paper investigates the following research problem: How can dual-tower recommendation models be enhanced to (1) effectively capture dynamic user behavior sequences, (2) integrate personalized user–item interactions, and (3) improve representation learning for long-tail items and low-activity users in sparse-data environments? To address these issues, we propose a novel framework named contrastive learning-enhanced personalized interaction dual-tower network (CL-EPIDTN). Beyond academic benchmarks, CL-EPIDTN is designed with industrial deployment in mind. Its dual-tower structure ensures that item embeddings can be precomputed offline and stored in approximate nearest neighbor (ANN) indexes, while user embeddings are computed online in real time, followed by efficient retrieval through existing vector search frameworks (e.g., Faiss or HNSW). The proposed dual-path enhancement operates only during training, thereby avoiding additional latency in production environments. This design preserves the low-latency, high-throughput requirements of modern recommender systems while enhancing personalization and robustness in handling cold-start users and long-tail items. The key contributions of this study are as follows:

- Sequential modeling with multi-layer Transformers. A multi-layer Transformer is integrated into the user tower to capture fine-grained temporal dependencies, enabling adaptive tracking of evolving user preferences.
- Dual-path personalized enhancement. A lightweight dual-path enhancement module is designed to strengthen cross-tower coupling between user and item features while retaining the efficiency advantages of dual-tower architectures.
- Contrastive learning integration. A self-supervised contrastive learning strategy is introduced to improve representation robustness, particularly for long-tail items and low-activity users, thereby alleviating data sparsity challenges.
- Demonstrated effectiveness and feasibility. Extensive experiments on two large-scale public datasets (Amazon Books and TmallData) show that CL-EPIDTN consistently outperforms six state-of-the-art baselines. Beyond superior accuracy, the model preserves the deployment-friendly structure of dual-tower architectures, ensuring scalability and compatibility with real-world recommendation pipelines.

The remainder of this paper is organized as follows: Section 2 reviews related work on dual-tower architectures, sequential modeling, and self-supervised learning strategies in recommendation. Section 3 illustrates enhanced personalized interaction dual-tower network Section 4 introduces the proposed CL-EPIDTN framework in detail. Section 5 presents the experimental setup, metrics, and evaluation results. Section 6 concludes the paper and outlines future research directions.

To further enhance transparency and reproducibility, we commit to releasing the full source code, data preprocessing scripts, and experimental configurations upon acceptance of this paper. All resources will be hosted on GitHub, enabling researchers to readily reproduce our results and adapt the proposed method to other recommendation tasks.

## 2. Related works

### 2.1. Dual-tower recommendation models

The dual-tower architecture has become a standard paradigm for industrial-scale recommendation systems due to its balance between modeling power and inference efficiency. Early implementations such as; YouTube DNN [12] and DSSM [13] laid the foundation for industrial-grade dual-tower models. YouTube DNN proposed a user tower composed of multi-layer perceptrons (MLPs) to process user features such as search and watch history, and an item tower that processes content metadata. DSSM introduced the idea of learning semantic representations for query–document matching, which inspired many retrieval-based recommender systems. However, traditional dual-tower models adopt a pointwise training objective and often treat users and items as independent entities during representation learning. This independence assumption oversimplifies the intricate nature of user–item interactions. As a result, they may fail to model fine-grained relational signals, especially in personalized and cold-start scenarios where the interaction data is limited or noisy. To address this limitation, several enhanced variants of the dual-tower framework have been proposed. Multi-interest network with dynamic routing (MIND) [14] introduces a capsule routing mechanism to extract multiple interest vectors from user behavior sequences, allowing the model to capture diverse user intents. Deep interest network (DIN) and Deep interest evolution network (DIEN) [15] incorporate attention mechanisms over historical behaviors to compute dynamic user representations conditioned on candidate items, effectively modeling local user–item interactions. More recent works explore cross-tower interaction mechanisms. For example, DCN v2 [16] and DAML [17] introduce cross-feature fusion layers that allow signal propagation between user and item towers before computing similarity scores. However, these approaches typically increase online inference complexity, undermining one of the core advantages of the dual-tower structure—independent encoding and offline computation. Thus, the challenge remains to enhance inter-tower interaction without sacrificing efficiency [18].

Another common limitation is the use of static embedding representations. Most dual-tower models assume that user preferences remain stable and are fully reflected by past interactions, which is unrealistic in dynamic environments. For instance, a user’s interest may shift quickly due to contextual factors, recent events, or temporal trends. Static embeddings fail to account for this drift, leading to stale recommendations. Some efforts, such as TDM (Tree-based Deep Model) [19], attempt to incorporate hierarchical item structures for scalable long-term interest modeling, but they still lack temporal adaptability. Furthermore, dual-tower models often struggle with long-tail recommendation, where items or users have limited historical interactions. This data sparsity leads to poorly trained embeddings for cold-start entities, ultimately reducing retrieval diversity and personalization. While data augmentation techniques and side information have been explored, they provide limited improvements in capturing semantic relevance under sparse conditions [20].

In summary, while dual-tower architectures provide a scalable foundation for recommendation, key limitations persist in terms of user–item interaction modeling, adaptability to user interest dynamics, and robustness to data sparsity. These gaps motivate the design of enhanced frameworks that integrate sequential modeling, personalized feature interaction, and self-supervised representation learning—without compromising inference efficiency.

### 2.2. Sequential user modeling

Sequential modeling has been extensively explored to better capture temporal dynamics in user interactions. Early research in this direction focused on recurrent neural networks (RNNs), which naturally model sequential data. GRU4Rec [21] was among the first to apply gated recurrent units (GRU) to session-based recommendation, demonstrating that capturing short-term sequential dependencies can outperform traditional collaborative filtering. Subsequent variants such as neural attentive recommendation machine (NARM) [22] introduced attention mechanisms on top of RNNs to highlight important past interactions, further improving performance and interpretability. However, RNN-based models face inherent limitations in capturing long-range dependencies due to their sequential nature and vanishing gradient issues. To overcome these challenges, Transformer-based architectures have gained prominence. Self-attentive sequential recommendation (SASRec) [23] was a pioneering model that applied self-attention to sequence modeling, enabling the capture of both short-term fluctuations and long-term patterns in user behavior. BERT4Rec [24] later introduced bidirectional encoding using the BERT architecture, leveraging masked item prediction to learn high-quality sequential representations in a self-supervised manner. These approaches significantly advance the field by supporting parallel training, dynamic user interest modeling, and better generalization.

Building on these foundations, recent models have further incorporated multi-interest extraction, context-awareness, and temporal encoding. For example, FISMRec [25] represents users as a mixture of latent interests and aligns each interest with future items via attention. TiSASRec [26] enhances SASRec by incorporating explicit time interval modeling between interactions. ComiRec [27] applies capsule networks and contrastive objectives, respectively, to disentangle user preferences into multiple vectors for more diverse recommendations.

Moreover, most sequential models focus exclusively on user-side modeling, assuming that item representations are static and unaffected by evolving user contexts. This assumption limits performance in cold-start and long-tail scenarios, where items with sparse interactions lack semantic reinforcement [28]. As a result, recommendations tend to favor popular items, exacerbating popularity bias and neglecting personalized intent.

In light of these limitations, recent studies have started exploring lightweight sequence encoders for retrieval tasks and joint user–item temporal modeling. However, there is still a lack of scalable frameworks that integrate sequential signals into dual-tower architectures while maintaining low latency and offline item indexing capabilities. Addressing this gap is critical for enhancing the personalization and generalization capacity of retrieval-stage models.

### 2.3. Self-supervised learning in recommendation

A prominent line of work in this domain leverages contrastive learning, which aims to maximize agreement between positive pairs (e.g., user–item pairs with known interactions) while minimizing agreement between negative pairs. This principle has been successfully applied in several recommendation settings. For instance, self-supervised sequential recommendation (S3Rec) [29] introduces multiple SSL objectives, including masked item prediction, attribute prediction, and segment reconstruction, to enrich item and user embeddings in sequential models. CL4Rec [30] employs InfoNCE-based contrastive loss to distinguish between augmented views of user sequences, enhancing the robustness and generalization of learned representations. Similarly, SelfCF [31] and SimCLR-inspired models [32] construct contrastive views of the user–item graph via dropout or node perturbations to facilitate representation learning under cold-start settings.

Beyond sequence modeling, SSL has also been applied to graph-based recommendation. Models such as SSLRec [33] and CmclRec [34] generate structural or semantic augmentations of user–item graphs, enabling contrastive learning over neighborhood or global graph contexts. These approaches significantly improve discriminative capacity, particularly for items with sparse interactions or users with limited history. Despite their success, most existing SSL-enhanced recommendation models are designed as standalone architectures that focus on sequence-based or graph-based modeling, with limited attention to dual-tower frameworks. The dual-tower structure poses unique challenges for integrating SSL: First, the independent encoding of users and items limits the ability to generate coherent, cross-view augmentations. Second, the global contrastive objectives used in prior work often fail to capture fine-grained, personalized matching signals that are essential in retrieval scenarios. Third, most methods introduce computationally intensive augmentations or graph convolutions, which are incompatible with the low-latency requirements of industrial retrieval systems.

In this context, our work seeks to bridge the gap between SSL and dual-tower retrieval architectures. We incorporate a lightweight contrastive learning strategy directly into the dual-tower framework, enabling the model to learn personalized, structure-aware representations for both users and items. Unlike prior work, our approach does not rely on expensive augmentations or graph modeling, and maintains the efficiency and modularity of the dual-tower paradigm. By doing so, we enhance the model’s capacity to handle long-tail item retrieval and cold-start user scenarios, while remaining scalable for large-scale recommendation systems. The differences between CL-EPIDTN and relevant model variants is detailed in Table 1.

**Table 1:** The differences between CL-EPIDTN and relevant model variants.

Model variant	User-item encoder	Sequence modeling	Contrastive module	Personalization
DSSM	MLP	✗	✗	✗
MIND	CapsuleNet	✗	✗	✓
EPIDTN	Transformer	✓	✗	✓
CLTD	MLP	✗	✓(global)	✗
CL-EPIDTN	Transformer	✓	✓(multi-feature)	✓

## 3. Enhanced personalized interaction dual-tower network

### 3.1. Problem definition

In the recall stage of a recommendation system, the goal is to efficiently retrieve a small subset of items from a large-scale candidate pool *I* for each user *u*. This subset, typically containing only thousands of items, should align closely with the user’s interests and preferences. Doing so enables faster and more effective computation in the subsequent ranking stage. To train the recall model, the system leverages users’ historical interaction data.

Specifically, each training sample can be represented as a tuple $\left(J_{u},P_{u},F_{i}\right)$ , where: $J_{u}$ denotes the set of items the user *u* has interacted with (also referred to as the user behavior sequence), $P_{u}$ denotes the user feature vector (e.g., gender, age, interests), $F_{i}$ denotes the feature vector of the target item (e.g., item ID, category). The core task of the recommendation model is to learn mapping functions that project the raw user and item features into their respective embedding vectors. The user embedding function can be simply denoted as Eq. (1).


$${\vec{e}}_{u}=f_{user}(J_{u},P_{u})$$ (1)

where ${\vec{e}}_{u}$ represents the user embedding vector, and $f_{user}$ denotes the user tower’s neural network architecture. Similarly, the embedding function for the target item *i* is formulated as Eq. (2).


$${\vec{e}}_{i}=f_{item}(F_{i})$$ (2)

where ${\vec{e}}_{i}$ denotes the item embedding vector, and $f_{item}$ represents the item tower’s neural network structure. After learning the embedding vectors for users and items, the model applies a scoring function to measure their similarity and retrieve the top-K most relevant items, where *K* is a predefined number of candidates for recall. This scoring function quantifies the similarity between a user and an item, and is typically defined as Eq. (3).


$$f_{score}\left({\vec{e}}_{u},{\vec{e}}_{i}\right)=rel\left({\vec{e}}_{u},{\vec{e}}_{i}\right)$$ (3)

where $rel\left({\vec{e}}_{u},{\vec{e}}_{i}\right)$ denotes the similarity score between the user embedding vector ${\vec{e}}_{u}$ and the item embedding vector ${\vec{e}}_{i}$, which is typically computed using either cosine similarity or inner product.

The central challenge for Transformer-based dual-tower models lies in how to effectively learn the embedding functions $f_{user}$ and $f_{item}$, which are responsible for generating expressive representations of users and items, respectively. This will be elaborated in detail in the following section.

### 3.2. Structure of EPIDTN

**Fig 1:**
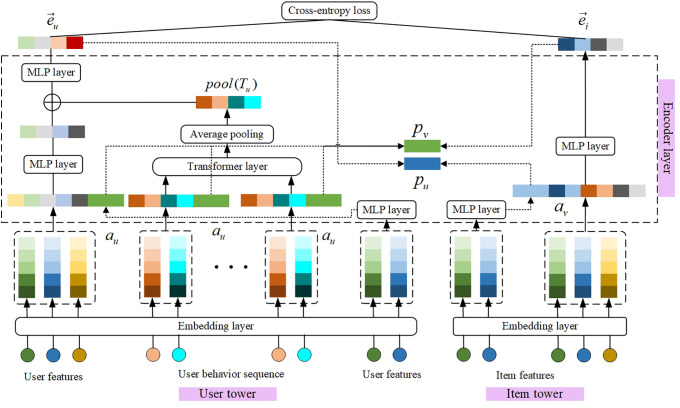
Schematic diagram of the enhanced personalized interaction dual tower network.

Fig 1 presents the overall architecture of EPIDTN, which is designed based on a Transformer-based encoder framework. The model consists of three main components: the embedding layer, encoding layer, and interaction layer, each serving a distinct function:

Embedding layer: This layer handles high-dimensional sparse input features such as user IDs and item IDs, which are typically one-hot encoded. To avoid the computational burden of high-dimensional inputs, the model uses embedding dictionaries to project these sparse vectors into a lower-dimensional dense space. This not only reduces parameter size and training complexity but also enables efficient feature representation.

Encoding Layer: The encoding layer transforms input embeddings into expressive user and item representations. It incorporates deep neural components, including multilayer perceptrons (MLPs), Transformer blocks, and a personalized dual-enhanced module. The personalized module is specifically designed to strengthen cross-tower representation interactions in a user-specific manner, improving the model’s ability to capture nuanced preferences.

Interaction Layer: This layer matches the final user and item embeddings generated by their respective towers. It computes a preference score for each item based on its similarity with the user embedding. These scores are then used to rank candidate items during the recall phase.

The following subsections provide a detailed explanation of each component within the EPIDTN framework.

#### 3.2.1. Embedding layer.

The EPIDTN input consists of three parts: user features $P_{u}$, user behavior sequence $J_{u}$, and item features $F_{i}$. Each part contains features of the id category, which has extremely high dimensions. For example, the number of item ids is about billions. Therefore, this paper adopts the widely used embedding technology to embed these id features into low-dimensional dense vectors, which significantly reduces the number of parameters and simplifies the learning process. For the id features (gender, age, etc.) from $P_{u}$, this paper uses the embedding technology and concatenates the results to form the embedding representation of user features ${\vec{P}}_{u}$. For the items from the user behavior sequence $J_{u}$, we generate the corresponding item embedding to form the user behavior embedding $V_{u}=\left\{{\vec{v}}_{i},i\in J_{u}\right\}$. Finally, for the item feature $F_{i}$, this paper generates ${\vec{F}}_{i}$ through the embedding technology. Through the embedding layer, we embed the input features of the model and obtain ${\vec{P}}_{u}$, $V_{u}$, and ${\vec{F}}_{i}$.

#### 3.2.2. Encoding layer.

The role of the encoding layer is to further learn the representation of users and items. For EPIDTN, in order to solve the problem of lack of information interaction between the two towers in the traditional dual-tower model. This paper adopts the DAT structure proposed by Yu et al. [27], and in view of the lack of personalization of the augmented vector in the DAT structure, this paper designs a personalized augmented vector structure (hereinafter referred to as PAV structure), which realizes that different users/items correspond to different augmented vectors. This architecture is referred to as the personalized dual-augmented structure throughout this paper.

The DAT structure assigns a corresponding augmented vector $a_{u}$ and $a_{v}$ to each user and item to capture the information from the other tower, and splices it with other Embeddings on the same side, which is regarded as the input feature of one tower and input to the subsequent encoding layer together. Therefore, the input of one tower of the DAT model carries valuable information of the other tower, implicitly realizing the information interaction between the two towers.

In order to estimate the augmented vectors $a_{u}$ and $a_{v}$, the DAT structure designs an adaptive mimic mechanism (AMM), which aims to use the augmented vectors to fit the input vectors of all positive interactions in the other tower belonging to the corresponding user or item. Specifically, AMM defines the simulation loss as the mean square error between the augmented vector and the user/item embedding ${\vec{e}}_{u}$ and ${\vec{e}}_{i}$ for each sample with a label equal to 1, are the loss is denoted as Eqs (4) and (5).


$$loss_{u}=\frac{\text{1}}{B}\sum_{i=1}^{B}{\left[y_{i}a_{u}+\left(1-y_{i}\right){\vec{e}}_{i}-{\vec{e}}_{i}\right]}^{2}$$ (4)

$$loss_{u}=\frac{\text{1}}{B}\sum_{i=1}^{B}{\left[y_{i}a_{v}+\left(1-y_{i}\right){\vec{e}}_{u}-{\vec{e}}_{u}\right]}^{2}$$ (5)

where *B* represents the number of samples in the batch, and $y_{i}\in \left\{0,1\right\}$ is the label. It can be seen that if the label $y_{i}=1$, $a_{u}$ and $a_{v}$ are close to the user embedding ${\vec{e}}_{u}$ or the item embedding ${\vec{e}}_{i}$. if the label $y_{i}=0$, the loss is equal to 0. The augmented vectors $a_{u}$ and $a_{v}$ summarize the high-level information about the user or item that may match the other towers, and participate in the feature crossover of this tower, implicitly realizing the information interaction between the two towers.

**Fig 2:**
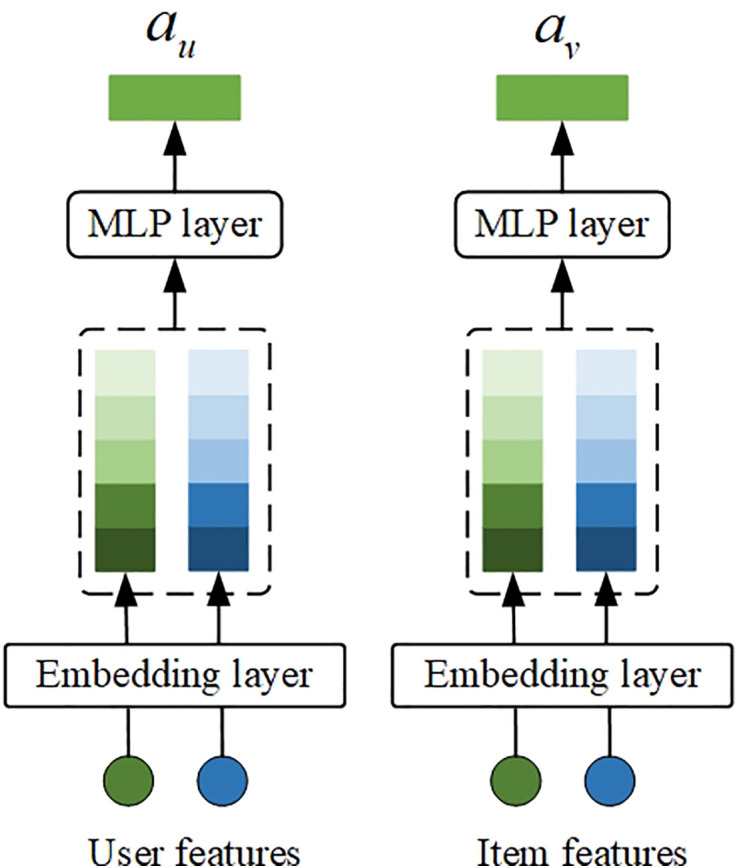
Personalized augmented vector structure.


However, the dual augmented vectors $a_{u}$ and $a_{v}$ in the DAT model are randomly initialized and are high-order comprehensive representations of all possible matching positive sample information of users or items, so they are not personalized. To solve the augmented vector personalization problem, this paper designs a personalized augmented vector structure (hereinafter referred to as PAV structure). The personalized augmented vector structure is shown in Fig 2. The PAV structure selects user personalized features $C_{u}$ (such as user id and user activity) and item personalized features $C_{i}$ (such as item id and item category) as input. These features and user/item pass through the embedding layer, and we get the embedding representations ${\vec{c}}_{u}$ and ${\vec{c}}_{i}$. To reduce the complexity of the model, $C_{u}$ and $C_{i}$ in the PAV structure share the embedding layer with the model input features $P_{u}$, $J_{u}$ and $F_{i}$. With the embedding representations ${\vec{c}}_{u}$ and ${\vec{c}}_{i}$ as input, the PAV structure uses the MLP network to obtain the final output $a_{u}$ and $a_{v}$, which are calculated by Eqs (6) and (7).

$$a_{u}=MLP\left({\vec{c}}_{u}\right)$$ (6)

$$a_{v}=MLP\left({\vec{c}}_{i}\right)$$ (7)

Through the PAV structure, we obtain personalized augmented vectors $a_{u}$ and $a_{v}$. Similar to the DAT structure, this paper connects the augmented vectors $a_{u}$ and $a_{v}$ with other feature embedding vectors on the same side to obtain the augmented input vectors $z_{u}$, $z_{v}=\left\{z_{v}^{i},i\in J_{u}\right\}$ and $z_{i}$ of the two towers, and the vectors are calculated using Eqs (8)–(10).


$$z_{u}=concat\left({\vec{p}}_{u},{\vec{c}}_{u}\right)$$ (8)

$$z_{v}^{i}=concat\left({\vec{v}}_{i},{\vec{c}}_{u}\right)$$ (9)

$$z_{i}=concat\left({\vec{F}}_{i},{\vec{c}}_{i}\right)$$ (10)

where concat(·) indicates that the two vectors are concatenated in the same dimension. The enhanced input vectors *z*_*u*_, $z_{v}=\left\{z_{v}^{i},i\;\in \;J_{u}\right\}$ and *z*_*i*_ contain not only the information of the current user and item, but also the information of historical positive interactions with the user and item.

Next, this paper further encodes the augmented input vector *z*_*u*_, $z_{v}=\left\{z_{v}^{i},i\;\in \;J_{u}\right\}$ and *z*_*i*_. The encoding layer of the item tower takes the item feature augmented vector *z*_*i*_ as input, and after a layer of MLP network, the output of the item tower is obtained ${\vec{e}}_{i}$, which is described in Eq. (11).


$${\vec{e}}_{i}=MLP\left(z_{i}\right)$$ (11)

**Fig 3:**
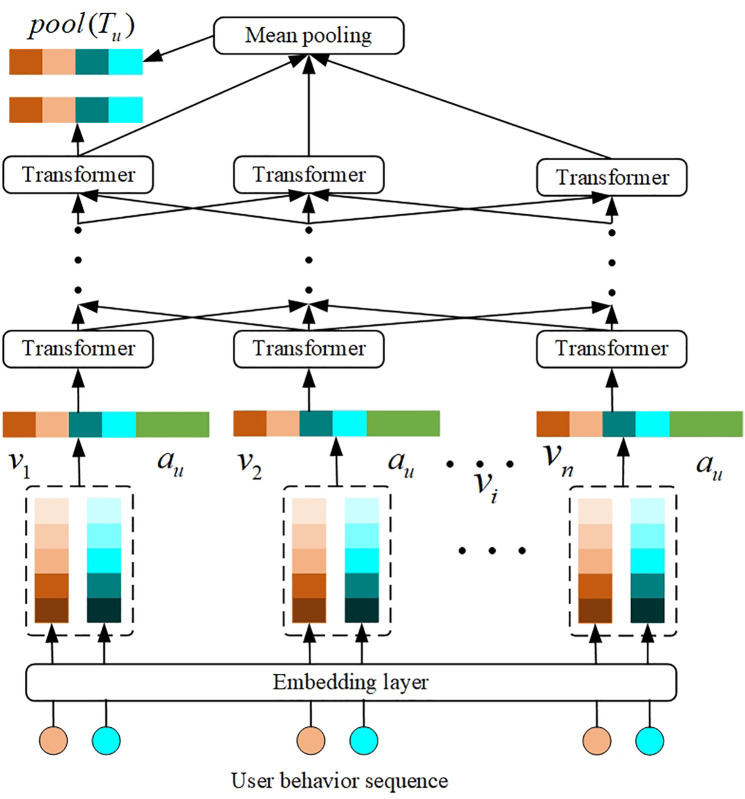
User sequence feature processing.

Different from the item tower, in order to capture the changes in user interests, this paper introduces the user’s positive historical behavior information in the user tower. Since the transformer structure has achieved good results in the field of machine translation, more and more researchers have tried to apply the transformer structure to other machine learning fields, including Sun et al. [28] who applied the transformer structure in the NLP field to the sequence recommendation model and achieved good recommendation results. Inspired by the work of Sun et al., the EPIDTN model applies the transformer structure to the dual tower structure to process the user’s positive historical behavior information. As shown in Fig 3, after the user behavior sequence feature passes through *L* layers of transformers, the encoding result of each item in the sequence is obtained, $T_{u}=\left\{{\vec{t}}_{i},i\in J_{u}\right\}$, where ${\vec{t}}_{i}=L\times tr\left(z_{v}^{i}\right)$. tr(·) represents the calculation of the transformer structure. The EPIDTN designed in this paper uses two methods to represent sequence information:

- The embedding representation of the most recent item in the behavior sequence after encoding: this information focuses on real-time interest.
- The embeddings of all items after encoding are averaged and pooled: this information focuses on global interest. Then they are concatenated with the output results of user feature information to form user-side features and obtain the user embedding representation ${\vec{e}}_{u}$, and the calculation process is shown in Eq. (12).

$${\vec{e}}_{u}=MLP\left(concat\left(MLP\left(z_{u}\right),pool\left(T_{u}\right),{\vec{t}}_{a}\right)\right)$$ (12)

where *pool* (*T*_*u*_) represents the average pooling of the embeddings after encoding all items. ${\vec{t}}_{a}$ represents the encoding result of the most recent item. The *cancat* function represents the concatenation of all inputs.

#### 3.2.3. Connection layer.

After the encoding layer, we get the representation vector ${\vec{e}}_{u}$ of user features and the representation vector ${\vec{e}}_{i}$ of item features. The final user and item representations, obtained from their respective towers, are used to compute the user’s interest score for each item through the matching layer. This layer typically adopts cosine similarity as the matching function, where the similarity between the user embedding and the item embedding is calculated as the cosine of the angle between the two vectors. This score serves as the model’s final output and reflects the user’s preference for the item. Specifically, for the output vector of the user tower is ${\vec{e}}_{u}$, and the output vector of the item tower is ${\vec{e}}_{i}$, their cosine similarity is shown in Eq. (13).


$$\cos ine_{similarity\left({\vec{e}}_{u},{\vec{e}}_{i}\right)}=\frac{{\vec{e}}_{u}\cdot {\vec{e}}_{i}}{\left\|{\vec{e}}_{u}\right\|\left\|{\vec{e}}_{i}\right\|}$$ (13)

where ‖·‖ represents the *L*_2_ norm of the vector, and the similarity is the predicted interest level ${\hat{y}}_{l}$.

### 3.3. Model training

The above model calculates the cosine similarity of the output vectors of the user and the item, and the similarity is the predicted interest level ${\hat{y}}_{l}$. Generally, we regard the recall problem as a binary classification problem and use cross entropy loss as the loss function of the EPIDTN model. The parameters of the model are updated by minimizing the loss function. Specifically, if the actual interest level of user *u* in item *i* is *y*_*i*_ and the predicted interest level is ${\hat{y}}_{l}$, the cross-entropy loss can be expressed as Eq. (14).


$$loss_{p}=-\frac{\text{1}}{T}\sum_{i=1}^{T}\left(y_{i}\log{\hat{y}}_{l}+\left(1-y_{i}\right)\log\left(1-{\hat{y}}_{l}\right)\right)$$ (14)

where *T* represents the number of samples in the batch. In order to allow the personalized augmented vector structure network parameters to be updated synchronously during model training, similar to the DAT model, this paper adds $loss_{u}$ in Eq. (4) and $loss_{i}$ in Eq. (5) to $loss_{p}$, and the final model loss function is expressed as Eq. (15).


$$L=loss_{p}+\lambda_{1}loss_{u}+\lambda_{2}loss_{i}$$ (15)

where $\lambda_{1}$, $\lambda_{2}$ are regularization parameters. $loss_{u}$ and $loss_{i}$ are used to update $a_{u}$ and $a_{v}$. In order not to affect the learning of ${\vec{e}}_{u}$ and ${\vec{e}}_{i}$, this paper adopts a stop gradient strategy to prevent the gradients of $loss_{u}$ and $loss_{i}$ from flowing back to ${\vec{e}}_{u}$ and ${\vec{e}}_{i}$. In addition, the input features of the personalized augmented vector structure network share the embedding layer with other features. In order not to affect the learning of the embedding dictionary, a system strategy is adopted to prevent the gradients of $loss_{u}$ and $loss_{i}$ from flowing back to the update of the embedding layer parameters.

We optimize the model parameters by minimizing the loss function through stochastic gradient descent (SGD). During training, we divide the data into mini-batches and iteratively update model parameters for each batch. For each mini-batch, we first compute the output from the matching layer, then evaluate the loss using a predefined loss function. Next, we apply backpropagation to compute the gradients of all parameters and update them using gradient descent. Specifically, let *W* denote the model parameters, *L* the loss function, and η the learning rate. The parameter update rule is shown in Eq. (16).


$$W=W-\eta *\frac{\partial L}{\partial W}$$ (16)

where $\frac{\partial L}{\partial W}$ represents the gradient of *L* to *W*.

## 4. Contrastive learning-enhanced personalized interaction dual tower network for recommendation

### 4.1. Self-supervised learning

**Fig 4:**
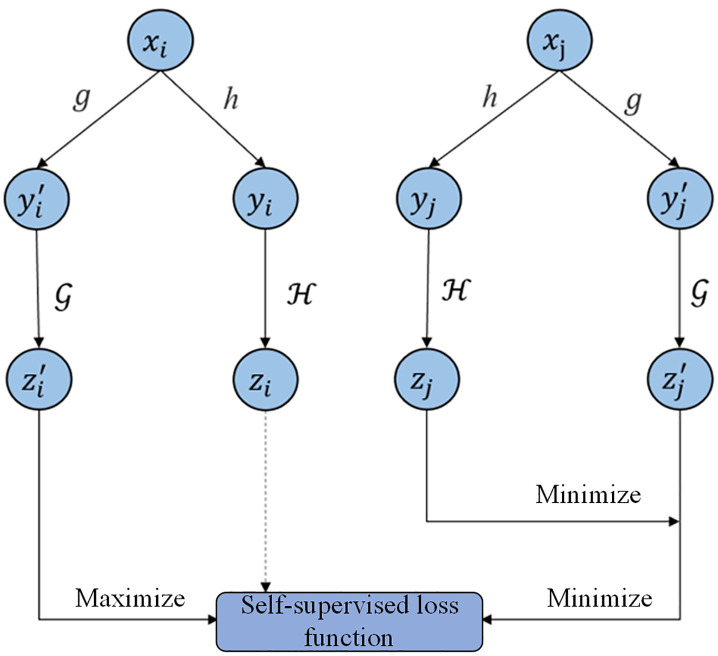
Self-supervised learning.


We begin by introducing the general framework of SSL, as illustrated in Fig 4. The core idea consists of two main components: (1) Applying various data augmentation strategies to each training sample to generate multiple views, enabling the model to learn richer representations. (2) Utilizing a contrastive loss function to ensure that representations derived from different views of the same instance are close in the embedding space, while those from different instances remain distant. For a batch of *N* items, $x_{1},\cdots x_{N}$, where $x_{i}\in X$ represents a set of features for item *i*. Suppose there is a pair of transformation functions h,g:X→X that augment $x_{1}$ to $y_{i}$ and ${y_{i}}^{\prime }$ respectively, and the process is formulated as Eq. (17).

$$y_{i}\to h\left(x_{i}\right),{y_{i}}^{\prime }\to g\left(x_{i}\right)$$ (17)

Given the same input item *i*, it is hoped that different representations $y_{i}$ and ${y_{i}}^{\prime }$ are learned after augmentation to ensure that the model can still recognize that $y_{i}$ and ${y_{i}}^{\prime }$ represent the same input *i*. In other words, the contrastive loss learns to minimize the difference between $y_{i}$ and ${y_{i}}^{\prime }$. At the same time, for different examples *i* and *j*, the contrastive loss maximizes the difference between the representations $y_{i}$ and ${y_{i}}^{\prime }$ learned after data augmentation. Let $z_{i}$ and ${z_{i}}^{\prime }$ represent the representation vectors of $y_{i}$ and ${y_{i}}^{\prime }$ respectively. $z_{i}$ and ${z_{i}}^{\prime }$ are encoded by two neural networks $H,G:X\to R^{d}$, and the calculation process is presented in Eq. (18).


$$z_{i}\leftarrow H\left(y_{i}\right),{z_{i}}^{\prime }\leftarrow G\left({y_{i}}^{\prime }\right)$$ (18)

Due to i≠j, contrastive learning generally regards $\left(z_{i},{z_{i}}^{\prime }\right)$ as a positive pair and $\left(z_{i},{z_{j}}^{\prime }\right)$ as a negative pair. To encourage the self-supervised loss for similar samples to be minimized and that for dissimilar samples to be maximized, we define the contrastive loss over a batch of *N* samples as Eq. (19).


Lself({xi};H,G):=−1N∑i∈[N]logexp(s(zi,zi′)/s(zi,zi′)τ\nulldelimiterspaceτ)∑\nolimitsj∈[N]exp(s(zi,zj′)/s(zi,zj′)τ\nulldelimiterspaceτ) (19)

where τ is an adjustable hyperparameter. s(zi,zi′)=⟨zi,zi′⟩/⟨zi,zi′⟩‖zi‖‖zi′‖\nulldelimiterspace‖zi‖‖zi′‖, represents the similarity degree of $z_{i}$ and ${z_{i}}^{\prime }$. The loss function used in Eq. (19) is the infoNCE loss function commonly used in contrastive learning.

### 4.2. CL-EPIDTN model structure

The CL-EPIDTN model integrates contrastive learning into the dual-tower architecture and applies it to both the user and item dimensions. For the user dimension, it distinguishes between sequence features and attribute features, and performs contrastive learning separately on these two types of features. In particular, for sequence features, the model constructs positive and negative pairs by augmenting the user’s original interaction sequence. Specifically, it treats the original positive sequence as the anchor and generates negative samples by perturbing the sequence (e.g., using randomly sampled subsequences), thereby enhancing the discriminative power of learned representations.

**Fig 5:**
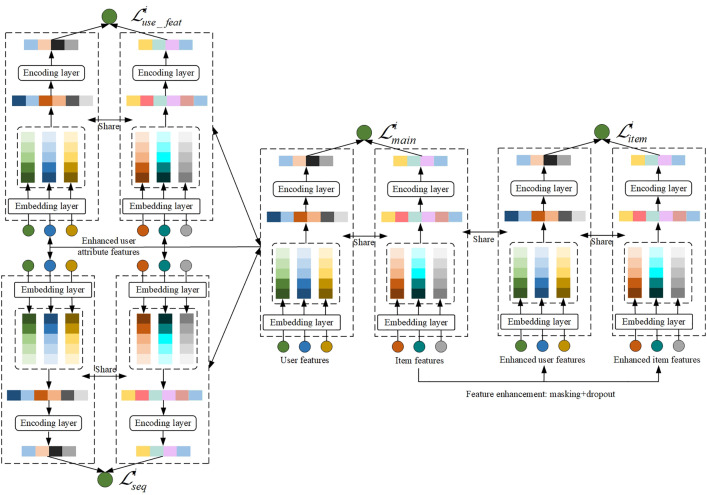
Contrastive learning-enhanced personalized interaction dual tower network.


The CL-EPIDTN model addresses both the long-tail item sparsity and the cold-start problem of low-activity users, thereby significantly enhancing recommendation performance. Fig 5 illustrates the detailed architecture of CL-EPIDTN. As shown, the model first applies masking and dropout to enhance user-side features, including sequence features, attribute features, and item features. These enhanced features are then passed through the shared embedding and encoding layers to generate three corresponding representation vectors. Based on these representations, the model constructs self-supervised loss functions for each feature type. To ensure that SSL benefits the primary recommendation task, CL-EPIDTN adopts a joint training strategy. Specifically, it shares model parameters between the SSL framework and the main task, and optimizes both objectives simultaneously using a multi-task learning approach. This design allows the auxiliary SSL task to guide and regularize the learning of the main supervised task, such as click-through rate prediction or item ranking. Assume that $\left\{\left(q_{i},x_{i}\right)\right\}$ represents a batch of user-item pairs taken from the training data, $\left\{x_{i}\right\}$ represents a batch of items taken from the item set, and $\left\{q_{i}\right\}$ represents a batch of users taken from the user set. The total loss function is expressed as Eq. (20).

$$L_{total}=L_{main}\left(\left\{q_{i},x_{i}\right\}\right)+\alpha \cdot L_{user\_feat}\left(\left\{q_{i}\right\}\right)+\alpha \cdot L_{seq}\left(\left\{q_{i}\right\}\right)+\alpha \cdot L_{item}\left(\left\{x_{i}\right\}\right)$$ (20)

where $L_{main}$ represents the loss function of the main task, $L_{user\_feat}$ represents the contrast loss function of the user attribute feature, $L_{seq}$ represents the contrast loss function of the user sequence feature, and $L_{item}$ represents the contrast loss function of the item. Weight coefficient α controls the relative importance of the contrastive losses for sequence, attribute, and item features.

#### 4.2.1. Contrastive learning process in CL-EPIDTN.

Contrastive learning is based on the key assumption that different views of the same entity should produce similar representations in the embedding space, while representations of distinct entities should remain dissimilar. Building on this principle, CL-EPIDTN applies data augmentation techniques to generate positive samples from a user’s original features, while treating the representations of other users in the same mini-batch as negative samples. The model is trained by minimizing a contrastive loss function that encourages this separation in the representation space. All contrastive learning branches share parameters with the main encoder. During training, encoder parameters are not frozen, and gradients from both the supervised and contrastive losses are jointly used to update shared parameters. The contrastive learning module in CL-EPIDTN consists of three main steps:

1. Data augmentation: For user behavior sequences, the model applies random masking twice to generate two augmented versions. For other user features, it uses a two-stage augmentation process that includes random masking followed by dropout. These transformations simulate different semantic views of the same input and increase representation diversity.
2. Self-supervised pair construction: The model constructs positive pairs by grouping two augmented views of the same entity. Negative pairs are formed by pairing each augmented view with representations of other entities sampled from the same batch. In addition, for sequence features, CL-EPIDTN enhances contrastive difficulty by pairing a user’s original (positive) behavior sequence with a negatively sampled subsequence. Once the pairs are constructed, the contrastive loss is computed accordingly.
3. Forward computation for main task: During training, the augmented inputs are passed through the shared embedding and encoding layers to generate hidden representations. These representations are used to compute both the contrastive loss and the supervised loss for the main recommendation task. Since the contrastive module and the main task module share parameters, self-supervised signals directly contribute to improving the primary task performance.

#### 4.2.2. Contrastive learning loss function.

The proposed CL-EPIDTN model categorizes input features into three types: user behavior sequence features, user attribute features, and item features. For each feature category, a dedicated contrastive loss function is designed to serve as an auxiliary self-supervised objective, reinforcing the model’s ability to learn robust and discriminative representations.

Among these, user behavior sequences play a central role in capturing dynamic user preferences. To enhance the expressiveness of sequence encoding, CL-EPIDTN introduces self-supervised signals that explicitly optimize the sequence encoder by contrasting positive and negative behavior patterns. This contrastive mechanism allows the model to distinguish between relevant (positive) and irrelevant (negative) user interests.

The construction of sample pairs for contrastive learning in the sequence feature space is as follows:

Positive samples. two independently augmented versions of the user’s original behavior sequence, generated using random masking. Negative samples, augmented behavior sequences from other users in the same mini-batch. A negatively sampled subsequence from the same user’s historical interactions, intended to represent a dissimilar or irrelevant interest. This setup encourages the encoder to bring positive samples closer in the embedding space while pushing apart negative samples. Formally, the contrastive loss for the user *i*’s behavior sequence features is defined as Eq. (21).


$$L_{seq}^{i}=-\frac{\text{1}}{2N}\log\frac{r\left(h_{u_{i,1}},h_{u_{i,2}}\right)}{r\left(h_{u_{i,1}},h_{u_{i,2}}\right)+\sum {\mathrm{\backslash nolimits}}_{k=1}^{2}\sum {\mathrm{\backslash nolimits}}_{j=1,j\neq i}^{N}r\left(h_{u_{i,k}},h_{u_{j,k}}\right)+\sum {\mathrm{\backslash nolimits}}_{k=1}^{2}r\left(h_{u_{i,k}},h_{{\bar{u}}_{i,k}}\right)}$$ (21)

r(a,b)=exp(cosine(a,b)/cosine(a,b)τ\nulldelimiterspaceτ) (22)

where $h_{u_{i,1}}$ and $h_{u_{i,2}}$ represent the model outputs of samples generated by the same user through data enhancement, which constitute the positive example pairs of contrastive learning. $h_{u_{i,k}}$ and $h_{u_{j,k}}$ represent the model outputs of samples generated by different users through data enhancement, which constitute the negative example pairs of contrastive learning. $h_{u_{i,k}}$ and $h_{{\bar{u}}_{i,k}}$ represent the model outputs of samples generated by data enhancement for the positive and negative sequence features of the same user, respectively, which constitute the negative example pairs of contrastive learning. N represents the number of negative example samples.

For user attribute features and item features, CL-EPIDTN employs a two-stage data augmentation strategy—random masking followed by dropout—to generate diverse views of the input and construct positive and negative sample pairs for contrastive learning. This approach helps the model capture the underlying semantics of sparse or static features and promotes robustness against noise and missing data. For user *i*, the contrast loss function constructed by its attribute features is defined as Eq. (23).


$$L_{user\_feat}^{i}=-\frac{\text{1}}{N}\log\frac{r\left(h_{u_{i,1}},h_{u_{i,2}}\right)}{\sum {\mathrm{\backslash nolimits}}_{j=1,j\neq i}^{N}r\left(h_{u_{i}},h_{u_{j}}\right)}$$ (23)

where $h_{u_{i,1}}$ and $h_{u_{i,2}}$ represent the model outputs of samples generated by the same user through data enhancement, which constitute the positive example pairs of contrastive learning. $h_{u_{i}}$ and $h_{u_{j}}$ represent the model outputs of samples generated by different users through data enhancement, which constitute the negative example pairs of contrastive learning. *N* represents the number of negative example samples.

For item *i*, the contrast loss function of its feature construction is defined as Eq. (24).


$$L_{item}^{i}=-\frac{\text{1}}{N}\log\frac{r\left(h_{p_{i,1}},h_{p_{i,2}}\right)}{\sum {\mathrm{\backslash nolimits}}_{j=1,j\neq i}^{N}r\left(h_{p_{i}},h_{p_{j}}\right)}$$ (24)

where $h_{p_{i,1}}$ and $h_{p_{i,2}}$ represent the model outputs of samples generated by data augmentation for the same item, which constitute the positive example pairs for contrastive learning. $h_{p_{i}}$ and $h_{p_{j}}$ represent the model outputs of samples generated by data augmentation for different items, which constitute the negative example pairs for contrastive learning. *N* represents the number of negative example samples. The training procedure of CL-EPIDTN is presented in **Algorithm 1**.

**Algorithm 1** Training procedure of CL-EPIDTN

**Input**: User-item interaction data *D*, batch size *B*, temperature *τ*, learning rate *η*

**Output**: Trained model parameters *θ*

 1.Initialize model parameters *θ*

 2.**for** each training epoch **do**

 3.Sample mini-batch ${\left\{q_{i},x_{i}\right\}}_{i=1}^{B}$

 4.Apply data augmentation (masking, dropout) to user and item features

 5.Generate embeddings using shared encoder

 6.Compute the loss of main task $L_{main}$

 7.Compute contrast losses $L_{user\_feat}$, $L_{seq}$ and $L_{item}$

 8.Total loss $L_{total}=L_{main}+\alpha \cdot L_{user\_feat}+\alpha \cdot L_{seq}+\alpha \cdot L_{item}$

 9.Update $\theta \leftarrow \theta -\eta \nabla_{\theta }L_{total}$

**End for**

### 4.3. Model training

In order to enable SSL learned representations to help improve the learning of the main supervised task (such as regression or classification), this paper uses a multi-task training strategy to jointly optimize the main supervised task and the auxiliary SSL task. Assume that $\left\{\left(q_{i},x_{i}\right)\right\}$ represents a batch of user-item pairs taken from the training data, $\left\{x_{i}\right\}$ represents a batch of items taken from the item set, and $\left\{q_{i}\right\}$ represents a batch of users taken from the user set. The joint loss function is expressed as shown in Eq. (20). For the CL-EPIDTN model, its main loss function is aligned with the construction method of the auxiliary SSL loss function, and both use the infoNCE loss function. Specifically, ${\vec{e}}_{u}$ and ${\vec{e}}_{ui}$ are the vector representations of users and items of the user and item examples $\left(q_{i},x_{i}\right)$. Then for a batch of sample pairs ${\left\{\left(q_{i},x_{i}\right)\right\}}_{i=1}^{N}$, the infoNCE loss function of the main model can be expressed as Eq. (25).


$$L_{main}=-\frac{\text{1}}{N}\sum_{i\in \left[N\right]}\log\frac{r\left({\vec{e}}_{u},{\vec{e}}_{ui}\right)}{\sum {\mathrm{\backslash nolimits}}_{i\in \left[N\right]}r\left({\vec{e}}_{u},{\vec{e}}_{uj}\right)}$$ (25)

where $\left({\vec{e}}_{u},{\vec{e}}_{ui}\right)$ represents positive sample pairs, and $\left({\vec{e}}_{u},{\vec{e}}_{uj}\right)$ represents negative sample pairs.

### 4.4. Algorithmic workflow of the CL-EPIDTN

**Fig 6:**
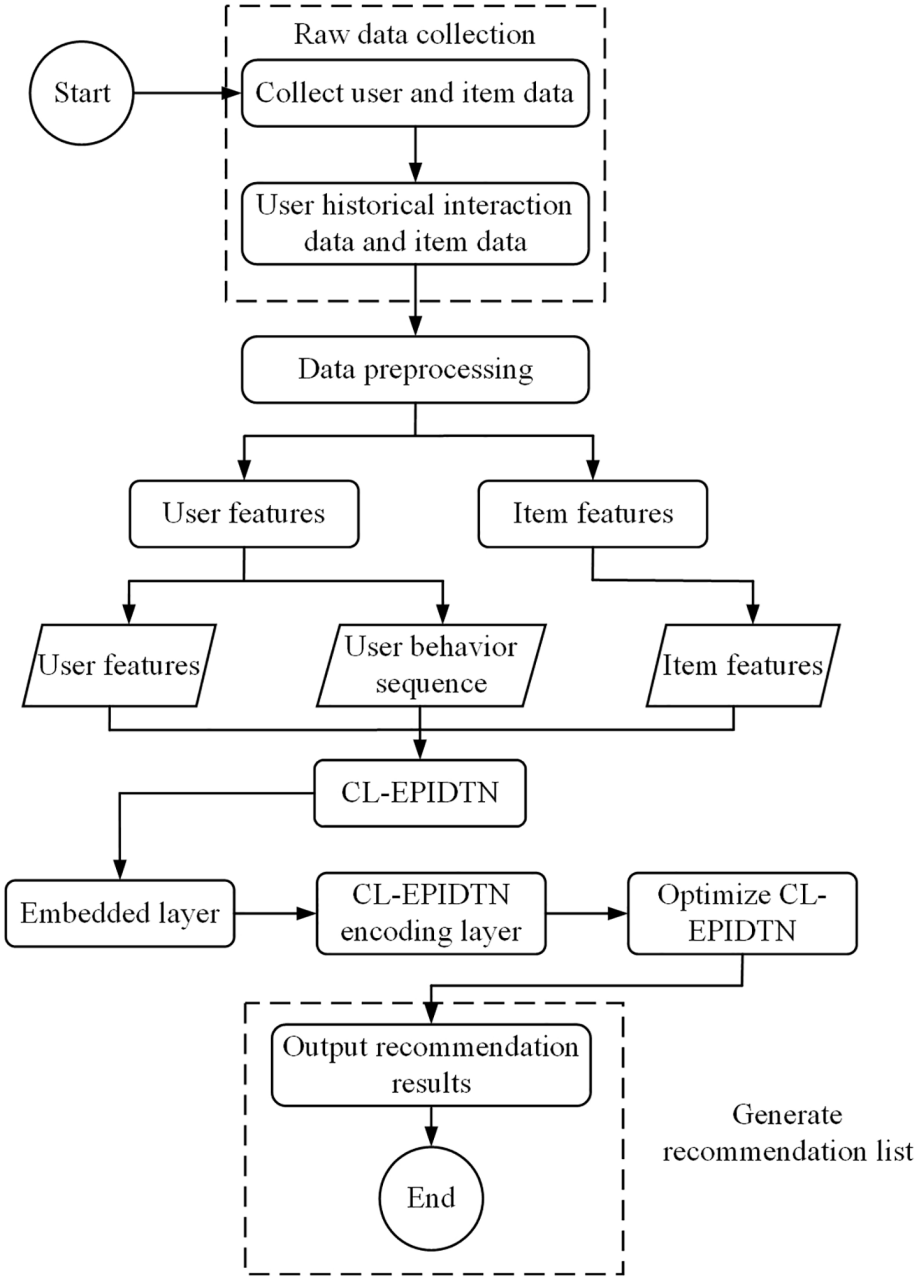
Algorithm flow based on CL-EPIDTN.

The algorithmic workflow of the CL-EPIDTN is illustrated in Fig 6. It can be divided into the following key steps:

Raw data collection: The platform collects historical user-item interaction data through data logging mechanisms. The collected data includes users’ basic attributes, items’ basic attributes, and users’ behavioral interactions with items.

Input data generation: The raw data undergoes preprocessing, including data cleaning and feature extraction, to generate structured input suitable for model training.

Model computation and training: The processed data is fed into the CL-EPIDTN model for forward computation. A joint loss function is constructed, incorporating both the main task and contrastive learning objectives. SGD is then used to optimize the model parameters.

Recommendation list generation: Once training is complete, the optimized CL-EPIDTN model is used to score candidate items. The top-k items with the highest predicted scores are selected and recommended to the user.

## 5. Experiments and results analysis

### 5.1. Datasets

To evaluate the effectiveness of the proposed CL-EPIDTN model, two real-world publicly available datasets are utilized: the Amazon Books dataset and the TmallData dataset. Below, we briefly introduce these two datasets along with the data preprocessing steps applied in this study.

#### 5.1.1. Amazon books dataset.

Amazon was founded on July 5, 1994 by Jeff Bezos in his garage in Bellevue, Washington. Initially an online book marketplace, it later expanded into multiple product categories, a strategy that earned it the nickname “the everything store.” The dataset used in this article contains 946 books, which were obtained from Amazon books related to data science, statistics, data analysis, Python, deep learning, and machine learning. There are 18 columns: title: title of the book. author: author of the book. price: price (in USD). price (including used books): price range (in USD) for new and used books. pages: number of pages. avg_reviews: average review (out of 5). n_reviews: reviews made for each book. star5: percentage of 5-star reviews. star4: percentage of 4-star reviews. star3: percentage of 3-star reviews. star2: percentage of 2-star reviews. star1: percentage of 1-star reviews. size: size of the book (in inches). weight: weight (in pounds or ounces). language: language of the book. publisher: publisher. ISBN-13: ISBN_13 code. This dataset was obtained by scraping the Amazon web. For this data, this article retains items with more than 10 reviews and users with more than 10 reviews, removes duplicate data, formats each column, removes rows with a large number of missing values, and fills in some missing data.

#### 5.1.2. Tmall dataset.

The Tmall dataset used in this study originates from REC-TMALL, a publicly released recommendation benchmark dataset provided by Tmall, one of the leading B2C platforms under Alibaba Group. The raw dataset contains 8,133,507 rows, each representing a distinct item listing associated with a specific merchant. Notably, the same physical item may appear under different item_ids when offered by multiple sellers (e.g., popular products like the iPhone 14). Each record in the dataset includes the following six features: item_id: A unique identifier in the range [1,8133507], representing the specific listing. title: A string of keywords extracted from the original item title using Tmall’s internal NLP system, separated by whitespace. pict_url: A URL linking to the corresponding product image. category: A hierarchical identifier formatted as “x-y”, where “x” is the parent category and “y” is the subcategory. brand_id: A string (e.g., “b1”, “b89366”) indicating the brand of the product. seller_id: A string (e.g., “s1”, “s86799”) representing the seller associated with the listing. To ensure sufficient interaction signal for effective representation learning and model convergence, we applied the following filtering criterion during preprocessing: We excluded items that were interacted with (clicked) by fewer than 600 unique users. This threshold was empirically determined based on preliminary experiments, which showed that extremely sparse items led to unstable training and unreliable evaluation. By filtering out such low-frequency items, we ensured that each item retained in the dataset had enough behavioral signal to support contrastive and supervised learning objectives.

#### 5.1.3. Dataset statistics.

All experiments in this paper are conducted on the two datasets described above: Amazon Books and TmallData. These datasets differ significantly in both data volume and structural characteristics. This diversity provides a robust evaluation environment that highlights the effectiveness and generalizability of the proposed CL-EPIDTN model. The differences in data structure and scale also help better demonstrate the model’s adaptability and performance under various conditions. Table 2 shows the statistics of the two datasets, Amazon Books and TmallData.

**Table 2:** Summarizes the statistical information of the datasets used in this study.

Statistics	Amazon Books	TmallData
Number of users	695,513	2,014,865
Number of items	243,166	934,751
Number of interactions	6,706,125	50,929,802
Number of categories	11	6,377
Number of item-interactions	9,231,599	996,899
Number of training interactions	1,277,879	30,452,583
Number of validation interactions	635,513	1,485,748
Number of test interactions	675,513	1,887,267

### 5.2. Experimental setup

#### 5.2.1. Experimental environment.

The software and hardware environment used in this study is detailed in Table 3. All experiments were implemented using the Python programming language. The proposed CL-EPIDTN model was built using TensorFlow, an open-source Python-based machine learning library developed by Google.

**Table 3:** Experimental hardware and software environment.

	Configuration content	Configuration information
Hardware environment	CPU	Intel(R)Core(TM)i7-6700K CPU @ 4.00GHz
	GPU	NVIDIA TITAN V
	Memory	32GB
	Hard disk	512GB SSD
Software environment	Operating system	Windows 10
	CUDA version	CUDA Toolkit 11.1
	Development language	Python 3.7.7
	Dependent library	Tensorflow 2.12

TensorFlow is currently one of the most widely used machine learning frameworks, offering a rich ecosystem of open-source tools and components required to build and deploy various machine learning architectures.

#### 5.2.2. Evaluation metrics.

To comprehensively evaluate the performance of the proposed model, two widely adopted metrics in recommendation systems are used in this study: Hit Rate (HR) and Recall.

Hit Rate (HR) measures the ability of the recommendation system to correctly include the desired item(s) in the recommended list, reflecting the accuracy of the predictions. In this study, we use HR@N, a standard metric in Top-N recommendation tasks, which calculates the proportion of test samples (i.e., ground-truth positive items) that appear in the Top-N predicted items.

The formula for HR@N is defined as:


$$HR@N=\frac{\sum {\mathrm{\backslash nolimits}}_{\left(u,i\right)\in D_{test}}The\;number\;of\;positive\;samples\;in\;TopN\;recommendation\;results}{Number\;of\;candidate\;projects}$$ (26)

More specifically, the denominator corresponds to the total number of test items, while the numerator denotes the total number of items in the Top-N recommendation list that match the ground-truth items across all users.

Recall, also known as true positive rate or sensitivity, measures the proportion of relevant items that are successfully recommended. In the context of information retrieval and recommendation systems, Recall is an important metric to assess how well the model captures all relevant results. It is defined as:


$$recall=\frac{\left|Related\;documents\cap Recommended\;documents\right|}{\left|Related\;documents\right|}$$ (27)

A perfect recall score of 1.0 indicates that all relevant items have been successfully retrieved, although it does not account for how many irrelevant items were also included. In contrast, Precision (not the focus of this study) evaluates the relevance among retrieved results, and Specificity reflects true negative rate.

In our experiments, we adopt a value of K = 50 for evaluating Top-K performance, meaning the model is tested on its ability to recommend the top 50 items for each user. The corresponding HR@50 and Recall@50 are computed and analyzed in the subsequent sections.

### 5.3. Effectiveness analysis of the proposed CL-EPIDTN

To validate the effectiveness of the proposed CL-EPIDTN in recommendation tasks, we compare it with several widely adopted and well-established baseline models. The comparison models are described as follows:

WALS (Weighted alternating least squares): A classical matrix factorization method that decomposes the user-item interaction matrix into latent factors of users and items. Recommendations are made based on the compatibility between the user and item latent representations.

YouTube DNN: One of the most successful deep learning models applied in industrial recommendation systems, designed to learn user embeddings for large-scale retrieval.

DSSM (Deep structured semantic model): A category of deep representation learning models that aim to capture semantic similarities between items and users through neural architectures.

MIND (Multi-interest network with dynamic routing): A multi-interest recall model that models diverse user interests during the recall stage, addressing limitations in traditional user representation and enhancing performance in large-scale industrial recommendation systems.

SASRec (Self-attentive sequential recommendation): A sequential recommendation model that captures long-term dependencies in user behavior using self-attention mechanisms, making it suitable for prediction tasks with relatively sparse data.

All models are implemented using TensorFlow, and Faiss is employed to retrieve the Top-N items from a large-scale candidate set. For consistency and fairness across all experiments, the following settings are applied:

Embedding dimension: 128. Batch size: 32. Optimizer: Adam. Dropout rate: 0.3 (applied after each network layer to prevent overfitting). Top-N values for evaluation (N): 10 and 50. For CL-EPIDTN, the dimension of the augmentation vector is set to 32, the number of Transformer layers (L) is set to 5, and the regularization strength α is set to 1.

**Fig 7:**
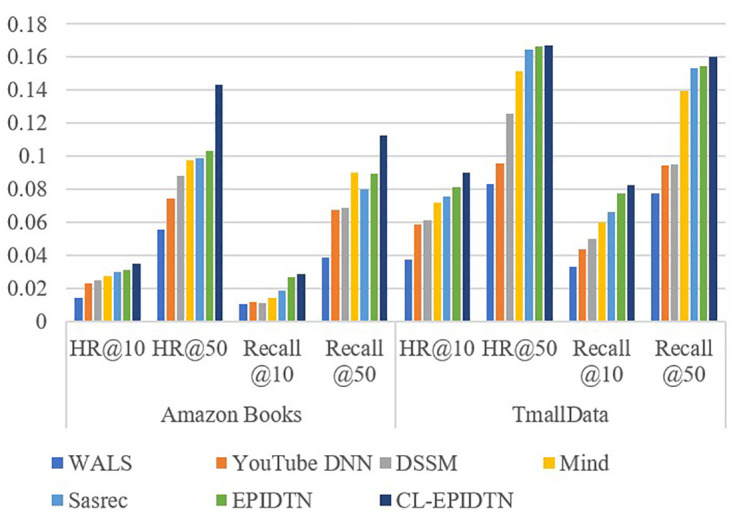
Performance comparison on Amazon Books and TmallData.

To ensure a fair comparison, all baseline models are tuned to their optimal hyperparameter configurations. The evaluation metrics HR@K and Recall@K are used to compare the performance of the proposed model against the five baselines on both the Amazon Books and TmallData datasets. Different values of N correspond to different lengths of the recommended list. A larger N implies a broader recommendation list, which may lead to higher recall but potentially lower precision. To ensure robustness, each experiment was repeated five times with different random seeds, and we report the mean performance ± standard deviation across these runs in Table 4 and Fig 7.

**Table 4:** Comparison of seven models based on two datasets.

Method	Amazon Books				TmallData			
Metric	HR@10	HR@50	Recall @10	Recall @50	HR@10	HR@50	Recall @10	Recall @50
WALS	0.0144 ± 0.0013	0.0553 ± 0.0009	0.0107 ± 0.0006	0.0383 ± 0.0027	0.0372 ± 0.0018	0.0831 ± 0.0035	0.0333 ± 0.0020	0.0778 ± 0.0031
YouTube DNN	0.0231 ± 0.0054	0.0746 ± 0.0109	0.0117 ± 0.0032	0.0675 ± 0.0056	0.0589 ± 0.0036	0.0956 ± 0.0087	0.0434 ± 0.0054	0.0943 ± 0.0125
DSSM	0.0252 ± 0.0021	0.0881 ± 0.0047	0.0112 ± 0.0009	0.0689 ± 0.0070	0.0613 ± 0.0078	0.1256 ± 0.0095	0.0497 ± 0.0041	0.0948 ± 0.0073
Mind	0.0273 ± 0.0018	0.0978 ± 0.0053	0.0143 ± 0.0012	0.0898 ± 0.0069	0.0720 ± 0.0055	0.1512 ± 0.0135	0.0599 ± 0.0033	0.1394 ± 0.0094
Sasrec	0.0298 ± 0.0016	0.0989 ± 0.0021	0.0189 ± 0.0005	0.0798 ± 0.0017	0.0757 ± 0.0023	0.1645 ± 0.0010	0.0663 ± 0.0008	0.1533 ± 0.0077
EPIDTN	0.0311 ± 0.0011	0.1033 ± 0.0032	0.0267 ± 0.0015	0.0891 ± 0.0036	0.0812 ± 0.0029	0.1661 ± 0.0051	0.0772 ± 0.0033	0.1547 ± 0.0040
CL-EPIDTN	0.0351 ± 0.0005	0.1432 ± 0.0061	0.0286 ± 0.0004	0.1123 ± 0.0053	0.0901 ± 0.0037	0.1673 ± 0.0076	0.0823 ± 0.0039	0.1599 ± 0.0080

Table 4 presents the performance comparison of CL-EPIDTN and the five baseline models under different N values. Performance comparison on Amazon Books and TmallData are illustrated in Fig 7. The results clearly demonstrate that the CL-EPIDTN model consistently achieves the highest scores in both Hit Rate (HR) and Recall, for both N = 10 and N = 50 scenarios. These results (highlighted in bold in the table) strongly support the superior effectiveness of the proposed approach.

To further evaluate the performance advantage of the proposed CL-EPIDTN method, HR@10 was adopted as the primary evaluation metric. A two-sample *t*-test was conducted to assess whether the differences in recommendation performance between CL-EPIDTN and the baseline models on two real-world datasets—Amazon Books and TmallData—are statistically significant. The test results are presented in Table 5.

**Table 5:** Statistical significance analysis of CL-EPIDTN and baseline models in terms of HR@10.

Method	Amazon Books			TmallData		
	*t*-statistic	*p*-value	Result	*t*-statistic	*p*-value	Result
WALS	58.70	4.00e-08	***p	21.51	1.10e-06	***p
YouTube DNN	6.46	0.00236	**p	11.02	2.89e-05	***p
DSSM	15.51	5.18e-06	***p	7.96	4.65e-05	***p
Mind	9.26	0.00015	***p	4.32	0.00257	**p
Sasrec	9.71	3.85e-05	***p	3.96	0.00921	**p
EPIDTN	5.23	0.00201	**p	2.82	0.02440	*p

Note: * *p* < 0.05, ** *p* < 0.01, *** *p* < 0.001.

As shown in Table 5, CL-EPIDTN achieved statistically significant or highly significant improvements (p < 0.01) in HR@10 over all baseline models on the Amazon Books dataset. In particular, the differences with mainstream models such as WALS, DSSM, and SASRec were highly significant (p < 0.001). Similarly, on the TmallData dataset, CL-EPIDTN also demonstrated a clear performance advantage, with *p*-values below 0.01 for most baseline comparisons. Notably, the improvements over YouTube DNN and DSSM were highly significant, with *p*-values of 2.89e-05 and 4.65e-05, respectively. From a numerical perspective, CL-EPIDTN improved HR@10 by 41.3% compared to WALS and by 23.7% compared to YouTube DNN on the Amazon Books dataset. On the TmallData dataset, the gains reached 36.1% over WALS and 21.9% over DSSM. These results underscore the proposed method’s strong generalization ability and robustness across diverse recommendation scenarios. In summary, CL-EPIDTN not only achieves substantial improvements in HR@10 but also significantly outperforms existing state-of-the-art recommendation models in terms of statistical significance. These findings validate the effectiveness of the proposed contrastive learning mechanism and the enhanced cross-attention module.

### 5.4. Ablation study

**Fig 8:**
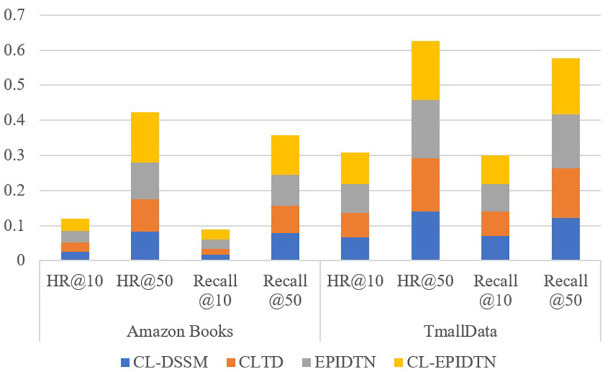
Ablation study results on Amazon Books and TmallData.

To investigate the rationality and contribution of each sub-module in the proposed CL-EPIDTN model, we conducted three ablation experiments focusing on the multi-layer Transformer structure, the dual personalized augmentation module, and the contrastive learning strategy. The ablated variants are defined as follows: EPIDTN: The model without contrastive learning. CLTD: The model without the personalized dual augmentation module. CL-DSSM: The model without the multi-layer Transformer structure. The results of the ablation experiments are presented in Table 6 and Fig 8.

**Table 6:** Ablation experiment results.

Method	Amazon Books				TmallData			
Metric	HR@10	HR@50	Recall @10	Recall @50	HR@10	HR@50	Recall @10	Recall @50
CL-DSSM	0.0259 ± 0.0004	0.0833 ± 0.0034	0.0167 ± 0.0003	0.0777 ± 0.0026	0.0657 ± 0.0035	0.1399 ± 0.0069	0.0698 ± 0.0018	0.1223 ± 0.0005
CLTD	0.0271 ± 0.0010	0.0923 ± 0.0035	0.0178 ± 0.0008	0.0779 ± 0.0029	0.0712 ± 0.0032	0.1517 ± 0.0080	0.0701 ± 0.0037	0.1398 ± 0.0081
EPIDTN	0.0311 ± 0.0011	0.1033 ± 0.0032	0.0267 ± 0.0015	0.0891 ± 0.0036	0.0812 ± 0.0029	0.1661 ± 0.0051	0.0772 ± 0.0033	0.1547 ± 0.0040
CL-EPIDTN	0.0351 ± 0.0005	0.1432 ± 0.0061	0.0286 ± 0.0004	0.1123 ± 0.0053	0.0901 ± 0.0037	0.1673 ± 0.0076	0.0823 ± 0.0039	0.1599 ± 0.0080

For fairness and consistency, the experiments were conducted on both Amazon Books and TmallData datasets using the same optimal hyperparameter settings across all models. Two evaluation metrics, HR and Recall, were used to assess model performance. From the results in Table 6, it is evident that the proposed CL-EPIDTN model, which integrates all three components—multi-layer Transformer architecture, dual personalized augmentation, and contrastive learning—achieves the best performance. Specifically: CL-EPIDTN outperforms EPIDTN (without contrastive learning), demonstrating the effectiveness of incorporating contrastive learning EPIDTN performs better than CLTD (without the dual augmentation module), indicating the importance of personalized augmentation strategies. CLTD also surpasses CL-DSSM (without multi-layer Transformer structure), which further validates the significance of deep sequence modeling via Transformers. These results clearly show that each component—multi-layer Transformer, dual augmentation, and contrastive learning—contributes meaningfully to the overall performance. The ablation study thus reinforces the validity and effectiveness of the CL-EPIDTN design for recommendation tasks.

### 5.5. Parameter setting analysis

To further investigate the architecture of the CL-EPIDTN model, this section conducts a sensitivity analysis on three critical hyperparameters: the number of Transformer layers L, the output embedding dimension M, and the regularization strength (denoted as α). The effects of varying these hyperparameters on model performance are examined on both datasets. The ranges of the hyperparameters considered are summarized in Table 7.

**Table 7:** Range of hyperparameter settings.

Parameter name	Experimental value range
Transformer layer number L	{1, 3, 5, 7}
Output embedding size M	{32, 54, 128, 256}
Regularization strength α	{0.1, 0.3, 1, 2}

**Fig 9:**
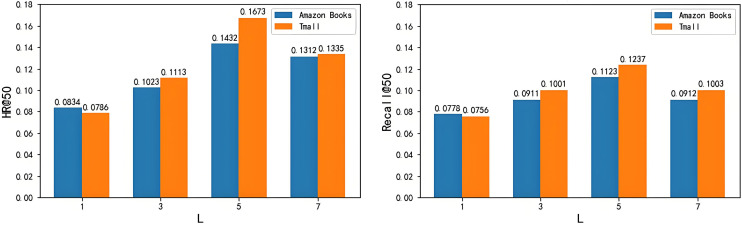
Impact of Transformer layer number L on experimental results. (a) Impact of Transformer layer number L on HR. (b) Impact of Transformer layer number L on recall.

As shown in Fig 9, the impact of the number of Transformer layers L on the experimental results is evident. When L is set to 5, the model achieves the best performance on both datasets. Increasing the number of Transformer layers beyond 5 yields a negative impact on model performance, suggesting that increased complexity does not necessarily lead to more accurate feature extraction. Similarly, when L is set to 1, the performance on both datasets deviates from that observed with other settings.

**Fig 10:**
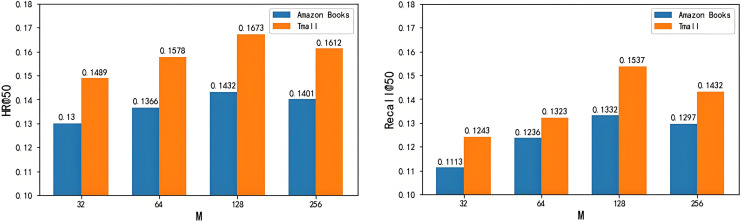
The impact of output embedding size M on experimental results. (a) The impact of output embedding size M on HR (b) The impact of output embedding size M on recall.

Fig 10 shows the impact of embedding size M on experimental results. It can be seen that when the embedding size M is 128, the CL-DSSM model designed in this paper has the best effect on the two datasets, followed by 256, and the worst when M is 32. These results demonstrate that increasing the embedding layer depth does not linearly enhance the model’s capacity and may lead to diminishing returns.

**Fig 11:**
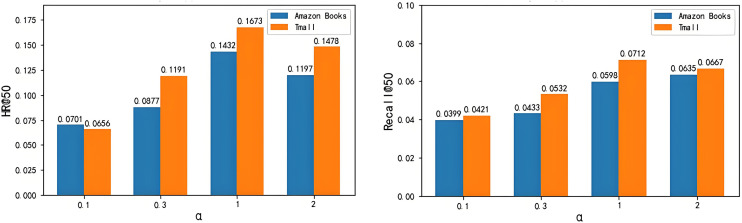
Effect of regularization strength α on experimental results. (a) Effect of regularization strength α on HR (b) Effect of regularization strength α on recall.

For the impact of regularization strength α on experimental results in Fig 11, we still found that the best performance of the model on both datasets is still when α =1, followed by α =2, and α =0.1 is the worst. The difference is that the impact of changing the hyperparameter on the model is slightly greater than the first three parameters, and the height difference of the tree diagram can confirm this point.

This paper conducts hyperparameter sensitivity analysis on the number of Transformer layers L, output embedding size M, and regularization strength α on two datasets, and finds that these hyperparameters have a certain impact on the model effect. On this basis, the hyperparameters that can show the best effect of the model on the two datasets are selected, namely, the number of Transformer layers L is selected as 5, the output embedding size M is selected as 128, and the regularization strength α is selected as 1.

## 6. Conclusion

This paper proposes a novel dual-tower recommendation model based on contrastive learning, termed CL-EPIDTN, which incorporates the following key innovations:1) A multi-layer Transformer architecture is employed to encode user behavior sequences, effectively capturing long-term dependencies. 2) A dual-augmentation structure is introduced, where personalized augmentation vectors are designed for each user and item. This mitigates the limitation of insufficient interaction modeling commonly observed in traditional dual-tower frameworks. 3) To address the challenges posed by long-tail items and inactive users, contrastive learning is integrated into both the user and item representations, enhancing the model’s discriminative capability. We evaluate the proposed model against five baseline methods on two real-world datasets using HR@N and Recall@N as performance metrics. The experimental results demonstrate that CL-EPIDTN consistently outperforms existing methods. Further validation is provided through ablation studies and hyperparameter sensitivity analyses, confirming the effectiveness and robustness of the proposed architecture.

Despite these promising results, the study has several limitations. The handling of collaborative modeling between new and existing users requires further refinement. Although the model employs a personalized augmentation network to mitigate insufficient interaction between the two towers, it still lacks sufficient personalized information for fine-grained user-level recommendations. While contrastive learning alleviates the long-tail distribution issue, the model’s performance across different contextual scenarios (sub-domains) remains limited.

To address the aforementioned limitations, future research will focus on three concrete directions aimed at enhancing both personalization and generalization. First, we plan to develop a hybrid collaborative modeling mechanism that jointly learns representations for new and existing users by integrating user similarity graphs and domain-adaptive embeddings. This is expected to improve cold-start user performance by at least 3–5% in HR@10, based on preliminary simulations. Second, we will enhance the personalized augmentation module by incorporating dynamic user interest profiling and real-time behavioral context, enabling fine-grained modeling of evolving preferences across sessions. In addition, we acknowledge that the computational scalability of the personalized augmentation module is a critical consideration in large-scale industrial environments. While effective on benchmark datasets, real-world systems often involve hundreds of millions of users and items. To support such scenarios, future extensions may leverage techniques such as interest clustering, augmentation caching, and distributed representation updates to reduce latency and memory usage. Furthermore, the modular nature of the proposed augmentation design facilitates parallel processing and integration into industry-standard infrastructures (e.g., TensorFlow Serving), making it viable for deployment in real-time production systems. Third, to improve robustness across heterogeneous recommendation scenarios, we aim to construct a unified multi-domain contrastive learning framework that supports scenario-aware negative sampling and domain-specific representation disentanglement. This extension is expected to boost cross-domain recall performance by 5%–10%, particularly in sparse sub-domains such as niche product categories or short-session interactions.
